# Tissue-engineered cornea constructed with compressed collagen and laser-perforated electrospun mat

**DOI:** 10.1038/s41598-017-01072-0

**Published:** 2017-04-20

**Authors:** Bin Kong, Wei Sun, Guoshi Chen, Song Tang, Ming Li, Zengwu Shao, Shengli Mi

**Affiliations:** 1 Macromolecular Platforms for Translational Medicine and Bio-Manufacturing Laboratory, Tsinghua-Berkeley Shenzhen Insititute, Shenzhen, 518055 P.R. China; 2 Biomanufacturing Engineering Laboratory, Graduate School at Shenzhen, Tsinghua University, Shenzhen, 518055 P.R. China; 3 Department of Mechanical Engineering, Drexel University, Philadelphia, PA USA; 4 Yantai SunPu Ruiyuan biological technology co., LTD., Yantai, 265500 P.R. China; 5 Shenzhen eye hospital, Shenzhen, 518000 P.R. China; 6 Tongji Medical Collage, Huazhong University Science & Technology, Wuhan, 430022 P.R. China; 7 Open FIESTA Center, Tsinghua University, Shenzhen, 518055 P.R. China

## Abstract

While Plastic Compressed (PC) collagen technique is often used to fabricate bioengineered constructs, PC collagen gels are too weak to be sutured or conveniently handled for clinical applications. To overcome this limitation, electrospun poly (lactic-co-glycolide) (PLGA) mats, which have excellent biocompatibility and mechanical properties, were combined with PC collagen to fabricate sandwich-like hybrid constructs. By laser-perforating holes with different sizes and spacings in the electrospun mats to regulate the mechanical properties and light transmittance of the hybrid constructs, we produced hybrid constructs with properties very suitable to apply in corneal tissue engineering. The maximum tensile stress of the optimal hybrid construct was 3.42 ± 0.22 MPa. The light transmittance of the hybrid construct after perforation was approximately 15-fold higher than before, and light transmittance increased gradually with increasing time. After immersing into PBS for 7 days, the transmittance of the optimal construct changed from 63 ± 2.17% to 72 ± 1.8% under 500 nm wavelength. The live/dead staining, cell proliferation assay and immunohistochemistry study of human corneal epithelial cells (HCECs) and human keratocytes (HKs) cultured on the optimal hybrid construct both demonstrated that the cells adhered, proliferated, and maintained their phenotype well on the material. In addition, after culturing for 2 weeks, the HCECs could form stratified layers. Thus, our designed construct is suitable for the construction of engineered corneal tissue.

## Introduction

Corneal trauma and ulceration, bacterial and viral infections, and heritable conditions are major contributors to corneal blindness, which affects over ten million individuals worldwide^1, 2^. Grafting allogenic corneal tissue is one of the primary therapies for serious diseases of the cornea because of its accessibility and immune privilege. However, there is a severe shortage of donor corneal tissue^3–5^ and many potential donor corneas are rejected because they do not meet standards^6^; thus, many researchers have attempted to fabricate corneal equivalents to replace pathologic corneal tissue^6, 7^. One method is to use human amniotic membrane (HAM), which is extensively used for the construction of damaged ocular surfaces and has been considered a gold standard scaffold for epithelial cell expansion^8–10^. HAM possesses the ability to reduce scarring and inflammation, to enhance wound healing, and to provide anti-fibrotic effects, yet this membrane has some drawbacks, such as risks of contamination and transmission of infectious diseases and biologic variability between donor tissues^11^. Another method is to use a fully bioengineered cornea with high biocompatibility and superior biological performance. The major difficulties for producing such a construct have been the generation of corneal substitute *in vitro* that exhibit strength and transparency equivalent to native cornea tissue^12^. Many approaches have been explored to construct three-dimensional (3-D) bioengineered corneal tissues *in vitro*. These strategies are to design biomimetic matrix systems for corneal tissue construction, such as via the hydrogel technique^13, 14^, prefabricated matrices^15, 16^, and decellularized corneal tissues^17–19^. However, these systems also have limitations, including insufficient mechanical strength and suturability of the hydrogel system, leading to further cross-linking, low production and immunogenicity of decellularized systems. Prefabricated matrices (e.g., nanofibrous and microporous scaffolds) have offered a feasible strategy to address these challenges in corneal tissue engineering.

Collagen is a suitable substrate for the fabrication of bioengineered corneal constructs because it has excellent biodegradability and biocompatibility, as well as low immunogenicity^20, 21^. However, the highly hydrated nature of conventional collagen gels renders them structurally weak and difficult to manipulate^22^. Several approaches have been taken to overcome the mechanical and geometrical drawbacks of collagen-based hydrogels^23–25^. A more practical and less labor-intensive method is the use of plastic compressed (PC) gels^26^; this technique was developed by Brown *et al*. and central to this process is to eliminate the majority of the water content of the gel. This presents a potentially ideal mechanism for controlling matrix and cell density, since only interstitial fluid is lost, increasing the cell and collagen density in a completely predictable manner dependent on the expelled fluid fraction^27^. However, although the mechanical properties of collagen gels are improved by compression, it still can’t meet the requirement of natural cornea and is too weak to be sutured or conveniently handled for clinical applications. Another novel method proposed by Connon *et al*. is the use of templates to instruct corneal stromal cells to create native extracellular collagen matrix, which had good structural, optical, mechanical, and biological properties, as a corneal tissue equivalent. They found that aligned collagen fibrils shown to be significantly thicker, denser, having a better mechanical properties than that of the randomly-oriented constituents^28^. The technique of electrospinning has attracted interests in fabricating biomimetic engineering functional corneal tissue due to the close structural resemblance of the constructs to native extra cellular matrix and its high surface area–to-volume ratio and good porosity, which provide support for cell adhesion and movement, proliferation and differentiation, as well as excellent mechanical properties, easy manipulation of fiber properties, great material handling, suturability for implantation, and scalable production. Many polymers can be produced into nanofibers via electrospinning^29–31^, and some electrospun constructs, such as those made of poly (lactic acid) (PLA), poly (ε-caprolactone) (PCL), and PLGA, have excellent mechanical properties that facilitate the simulation of natural tissue constructs, as well as clinical manipulation^32–34^.

To obtain a construct exhibiting good biocompatibility and mechanical properties, the electrospinning and PC collagen techniques were combined in this study. FDA-approved PLGA was chosen for the fabrication of the electrospun mat because of its compatibility, mechanical properties and degradation rate^35–39^. As mentioned above, producing a construct *in vitro* that exhibits strength and transparency equivalent to those of native corneal tissue is difficult. However, by post-processing the electrospun mat with laser perforation to create holes with different diameters and spacings, the strength and transparency of the mat, and thus those of the hybrid construct, can be regulated. This method can be used to create a construct with properties that are similar to those of natural corneal tissue. And the structure of the hybrid construct can realize the co-culture of two kinds of cells, within the PC collagen and on the surface of the hybrid construct. Thus, the combination of PC collagen and a laser-perforated electrospun PLGA scaffold was studied in this paper. In addition, we studied the activities of corneal cells (HCECs and HKs) on the designed hybrid construct and its potential for corneal tissue reconstruction.

## Materials and Methods

### Fabrication of aligned PLGA fibrous scaffolds

The PLGA (with a lactide: glycolide ratio of 50:50) solution (20% w/v) (molecular weight: 80000 g/mol, Sigma-Aldrich, UK) was prepared by dissolution in a chloroform/DMF mixture with a volume ratio of 90:10 at 50 °C under magnetic stirring for 3 h (analytical-grade chloroform and DMF, Sigma, Sweden). The spinning conditions were as follows: 18 cm needle collector distance; 12 kV voltage applied to a blunt, 23 gauge needle tip of a 5-mL syringe filled with the solution; 1 mL·h^−1^ flow rate; 300 rpm rotating speed, and 3 h spinning time. A detailed description of the electrospinning setup used to collect the aligned nanofibers has been previously published^40^. The humidity was adjusted to approximately 50%, and a temperature of 25 °C was maintained during the process. The newly formed electrospun mats were separated from the collector and vacuum dried at 50 °C for 24 h. The thickness of the fibrous scaffolds was determined by micrometer. Average fiber diameter and alignment degree were measured from scanning electron microscopy (SEM, S-4800 FESEM, HITACHI) images using Image Pro Plus (IPP) software.

### Picosecond laser perforation of aligned PLGA fibrous scaffolds

We used a 10 W picosecond laser machine (EP-IRPS10, Hans Laser) to perform laser perforation. For the *in vitro* study samples, through-holes were perforated in the aligned fibrous scaffolds. To obtain optimal mechanical properties and transparency, six samples with different hole diameters and spacings were made. The hole diameters (D) were 100, 150 and 200 μm, and the hole spacings (S) were 50 and 100 μm. The six samples were denoted as the following (D-S): 100–50 μm, 150–50 μm, 200–50 μm, 100–100 μm, 150–100 μm and 200–100 μm.

### Preparation of acellular, laser-perforated hybrid constructs

The collagen gel preparation and plastic compression were performed as previously described^27, 41, 42^. A volume of 4 mL of sterile rat-tail type I collagen (2.06 mg/mL protein in 0.6% acetic acid, First Link Ltd., West Midlands, UK) was mixed with 1 mL of 10× Eagle’s Minimum Essential Medium (Invitrogen Ltd., Paisley, UK). The mixture was neutralized with 1 M NaOH, cast into rectangular molds (33 mm × 13 mm × 8 mm), and allowed to undergo fibrillogenesis at 37 °C in a 5% CO_2_ incubator for 20 min, forming the first collagen layer. Then, the ethanol-sterilized, laser-perforated, electrospun PLGA mat was placed on the first collagen layer, and an additional 5 mL of collagen mixture solution was poured into the molds to form a second layer of collagen. Gel formation and polymerization were completed in the incubator within 30 min. The gels were placed on a blotting set consisting of nylon mesh (mesh size of w40 mm), stainless steel mesh (mesh size of w200 mm) and three sterile gauze pads. Additional nylon and stainless steel meshes were placed on top of the gel, and the construct was subjected to unconfined compression by a 120 g weight for 5 min, yielding the hybrid construct.

### SEM and mechanical properties of hybrid construct

Aligned electrospun constructs and hybrid constructs were dried by supercritical carbon dioxide extraction (5 × 5 min, 1 × 2 min). Samples were coated with a 20 nm-thick layer of platinum and examined by SEM at 3 kV.

The strain-stress properties of six laser-perforated hybrid constructs, control group (hybrid constructs without laser perforation) and acellular pig cornea were determined using tensile extension by a uniaxial load test machine (Electropuls E3000, INSTRON). Loading cycles were performed using a load sensor of 3000 N and a loading speed of 2 mm·min^−1^. Samples were prepared by incubating the samples in PBS (PH = 7.4) for 1 h (day 0), 7 and 21 days at 37 °C and then cutting them in a rectangular shape (30 mm × 5 mm) with the thickness 150–200 um. They were preconditioned by load-unload for 10 times under the same load level. The stress-stain curves were recorded, the maximum tensile strength and elongation at break were obtained. Three samples were measured for each construct allowing the mean and standard deviation to be calculated.

### Transparency test

The light transmission test involved soaking six hybrid samples, control hybrid constructs and acellular pig corneas in PBS at 37 °C for 0, 1, 3, 7 days, and then using a trephine to cut them into circles with a diameter of 6.5 mm, which is similar to the diameter of a well in a 96-well plate. These samples were then placed in 96-well plates with PBS; the blank control was PBS. The microplate reader (Multiskan Spectrum, purchased from Thermo Scientific) was then used to determine the light transmittance of the constructs under wavelengths in the range between 400 nm and 750 nm. The average of three measurements under each wavelength was calculated for each sample.

### Cell culture

Human corneal epithelial cells (HCECs)^43–48^ were obtained from the RIKEN BioResource Center (Tsukuba, Japan), and human keratocytes (HKs)^49, 50^ were donated by the He Eye Hospital (Shenyang, China). The HCECs were maintained in fresh medium consisting of Dulbecco’s modified Eagle’s medium (DMEM)-F12 (50:50, v/v Invitrogen) supplemented with 10% fetal bovine serum (FBS; HyClone), 100 U/ml penicillin and 100 μg/ml streptomycin (Invitrogen), recombinant human epidermal growth factor (10 ng/ml, Thermo Fisher) and bovine insulin (5 μg/ml, Thermo fisher). The HKs were maintained in Defined Keratinocyte-serum free medium (KSFM) (Gibco) with the addition of Defined Keratinocyte-SFM Growth Supplement and gentamicin (5 μg/ml, Amresco). The cells were cultured in an incubator at 37 °C with an atmosphere of 5% CO_2_. The cells were subcultured using trypsin (0.25%, Invitrogen) upon reaching approximately 80% confluence. The culture media were changed every 2–3 days.

### Cell-seeded hybrid constructs

Cells were seeded inside PC collagen gels by replacing the 0.5 mL of cell-free medium with 0.5 mL of HKs suspended (1.0 × 10^5^ cells·mL^−1^) in the neutralized collagen solution. HKs were seeded in both collagen layers. HCECs were seeded on top of the hybrid construct after plastic compression by dropping 0.1 mL of cell suspension (1 × 10^4^ cells) onto the construct surface and allowing the cells to attach overnight (humidified incubator, 37 °C, 5% CO_2_) before the addition of 0.1 mL of KSFM medium. After culturing for 7 days, the constructs were transferred to a 24-transwell (Costar, Corning Incorporated Life Sciences, Schipohl-Rijk, NL) to realize the air-lifting culture of HCECs for one week. The plates were incubated in an incubator at 37 °C with a humidified 5% CO_2_ atmosphere.

### Live/dead staining

A two-color fluorescence assay (LIVE/DEAD Assay, Molecular Probes, consisting of calcein, as a marker of viable cells, and ethidium homodimer, as a marker of dead cells, Invitrogen) was employed to determine HKs viability within the hybrid constructs immediately after the hybrid construct was produced, and to determine the HCECs viability after the cells incubating on the surface of the construct for 24 h. Each sample was washed in PBS 3 times before staining. Samples were stained for 15 min while avoiding light and washed 3 times in PBS after staining. A confocal microscope (Xcellence, Olympus) was used for image acquisition. Three different fields were counted for each sample.

### Cell proliferation assay

The Cell Counting Kit-8 (CCK-8, DOJINDO) was used to analyze the proliferation of HKs and HCECs on the hybrid construct according to the manufacturer’s instructions. Briefly, the hybrid construct with HKs cells in the PC collagen and the hybrid construct with HCECs inoculated on the top of it were washed with PBS three times, respectively. Then, 50 μL CCK-8 and 500 μL culture medium were added to each culture dish and incubated in the dark for 2 h at 37 °C. After incubation, 0.5 ml of the culture medium was transferred to a 96-well plate, and the optical density (OD) at 450 nm was immediately read. The constructs without cells were treated the same way, and the CCK-8 medium was used as the blank control. Three samples were tested for each group.

### Immunostaining of HKs and HCECs on the construct

PC collagen-PLGA hybrids with HKs and HCECs (the cell-seeding method was described in section “Cell-seeded hybrid constructs”) were divided into two groups, A and B. HKs were stained in group A, and HCECs were stained in group B. The constructs in the two groups were fixed with a solution of 4% paraformaldehyde at room temperature for 20 min. The samples were subsequently permeabilized in 0.1% Triton X-100 for 30 min and incubated with 1% (w/v) bovine serum albumin (Sigma) for 30 min to block non-specific binding. Samples in group A were then incubated for 1 h at 37 °C with the primary antibody against ALDH3A1 (1:50, Abcam) and then incubated for 1 h at 37 °C with FITC-labeled secondary antibody goat - anti -mouse (1:200, Abcam). Samples in group B were treated in a similar manner, except they were incubated with the primary antibody against cytokeratin 3 (CK3) (1:50, Abcam). Between each solution addition, the constructs were gently washed with PBS 3 times. Finally, group A, B were co-stained with DAPI (Thermo Fisher) and PI (Thermo Fisher) respectively and observed by confocal microscopy. The operations and observations were performed after 1, 3, and 7 days of culture.

### Immunofluorescent and histological staining of forzen sections

After culturing for 2 weeks, the constructs were embedded in optimal cutting temperature (OCT) (TissueTek, Sakura Finetek UK, Thatcham, United Kingdom), frozen in liquid nitrogen, and then cryosectioned. Prior to immunocytochemistry, each section (10 mm thick) was blocked using 5% bovine serum albumin (BSA) in 50 mM Tris-buffered saline (TBS; pH 7.2) containing 0.4% Triton X-100 for 60 min at room temperature. Sections were then incubated overnight at 4 °C with primary antibodies against CK3 (1:50, Abcam), diluted in 1% bovine serum albumin in TBS containing 0.4% Triton X-100. Fluorescein isothiocyanate (FITC)-labeled secondary antibodies goat - anti -mouse (1:200, Abcam) were used for 1 h at room temperature. Sections were were co-stained with PI (Thermo Fisher) and observed by confocal microscopy.

### Statistical analysis

All the results were presented as the mean ± standard deviation (SD). Statistical analysis was performed using two-way ANOVA with a Bonferroni post hoc test to determine the degree of significance. Statistical significance was defined as *p < 0.05.

**Figure 1:**
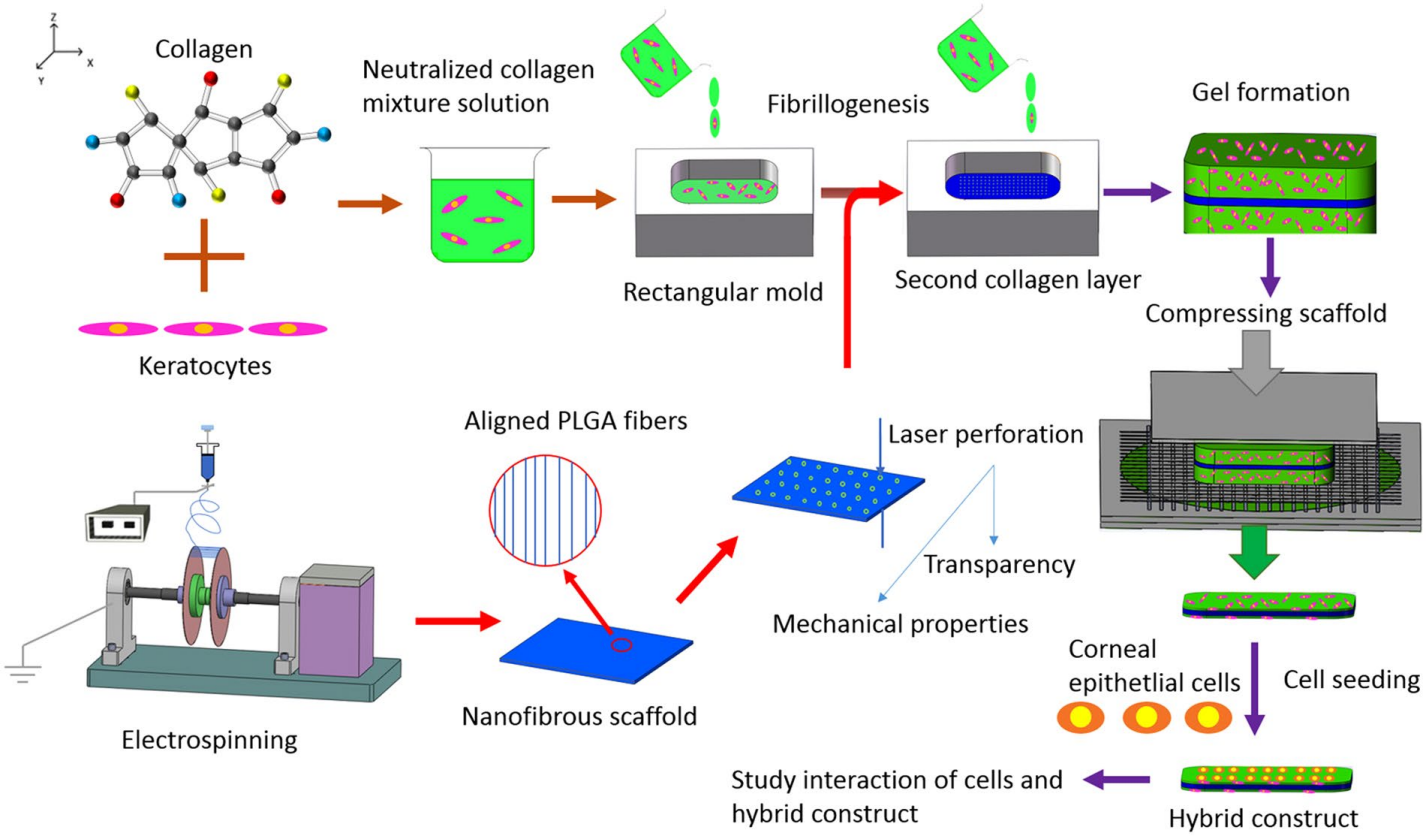
The schematic illustration of the experimental process.

The schematic illustration of the experimental process is shown in Fig. 1.

## Results

### Characterization of pure PLGA scaffolds and hybrid constructs

**Figure 2:**
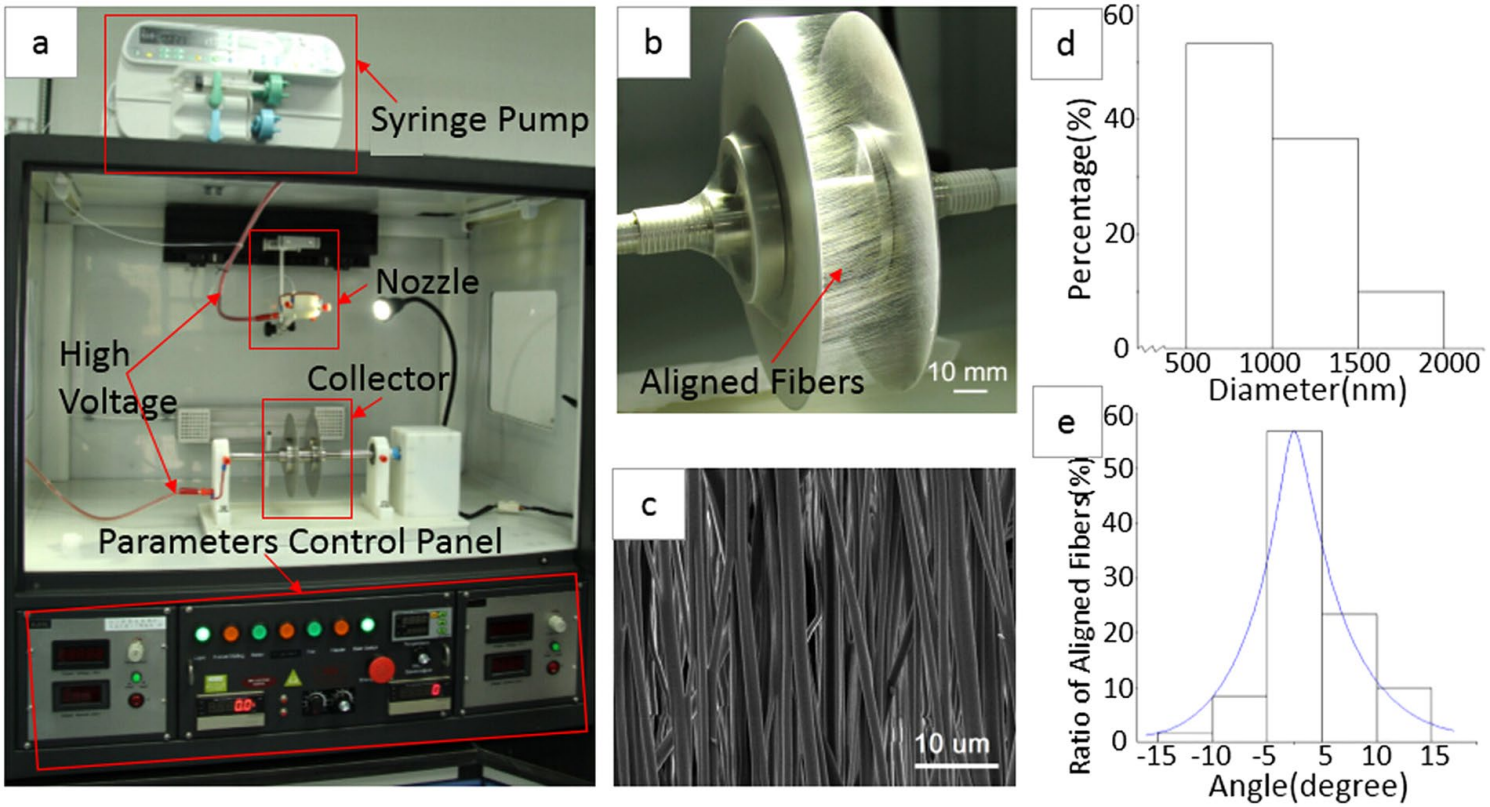
Electrospinning device and PLGA nanofibrous scaffold: (**a**) the main component of the device; (**b**) aligned fibers deposited between two parallel, thin plates; (**c**) an SEM image of a PLGA scaffold; (**d**) a histogram of fiber diameter; and (**e**) a histogram of fiber alignment.

The electrospinning device that we designed and used to produce aligned fibers is shown in Fig. 2a; the device consists of a solution supply system, a high-voltage power supply, and a fiber collector. The key component is the collector, i.e., two parallel thin plates, which was used to regulate the orientation of the electrostatic field, resulting in the deposition of aligned fibers between plates. As shown in Fig. 2b, the fibers were highly parallel on the collector. The diameter and degree of fiber alignment could be regulated by controlling electrospinning parameters, including the rotation speed of the collector shaft, the distance between the two plates, the distance between the needle and collector, and the voltage. The optimal parameters yielded the best degree of alignment^40^. Figure 2c shows an SEM image of electrospun PLGA fibers under the parameters mentioned in section “Fabrication of aligned PLGA fibrous scaffolds”. The thickness of the fibrous scaffold is 70 ± 10 um, which was determined by micrometer. The fibers were highly ordered and had diameters ranging from 500 nm to 2000 nm, as shown in Fig. 2d. The fiber alignment degree for samples was analyzed by the degree of deviation of the fibers from the standard angle using the method previously described^51^, and the alignment degree is represented in Fig. 2e by a histogram; 58% of the fibers were oriented within approximately ±5°, quantitatively demonstrating that the fibers were highly aligned. The data shown in Fig. 2d and e were obtained using IPP software.

**Figure 3:**
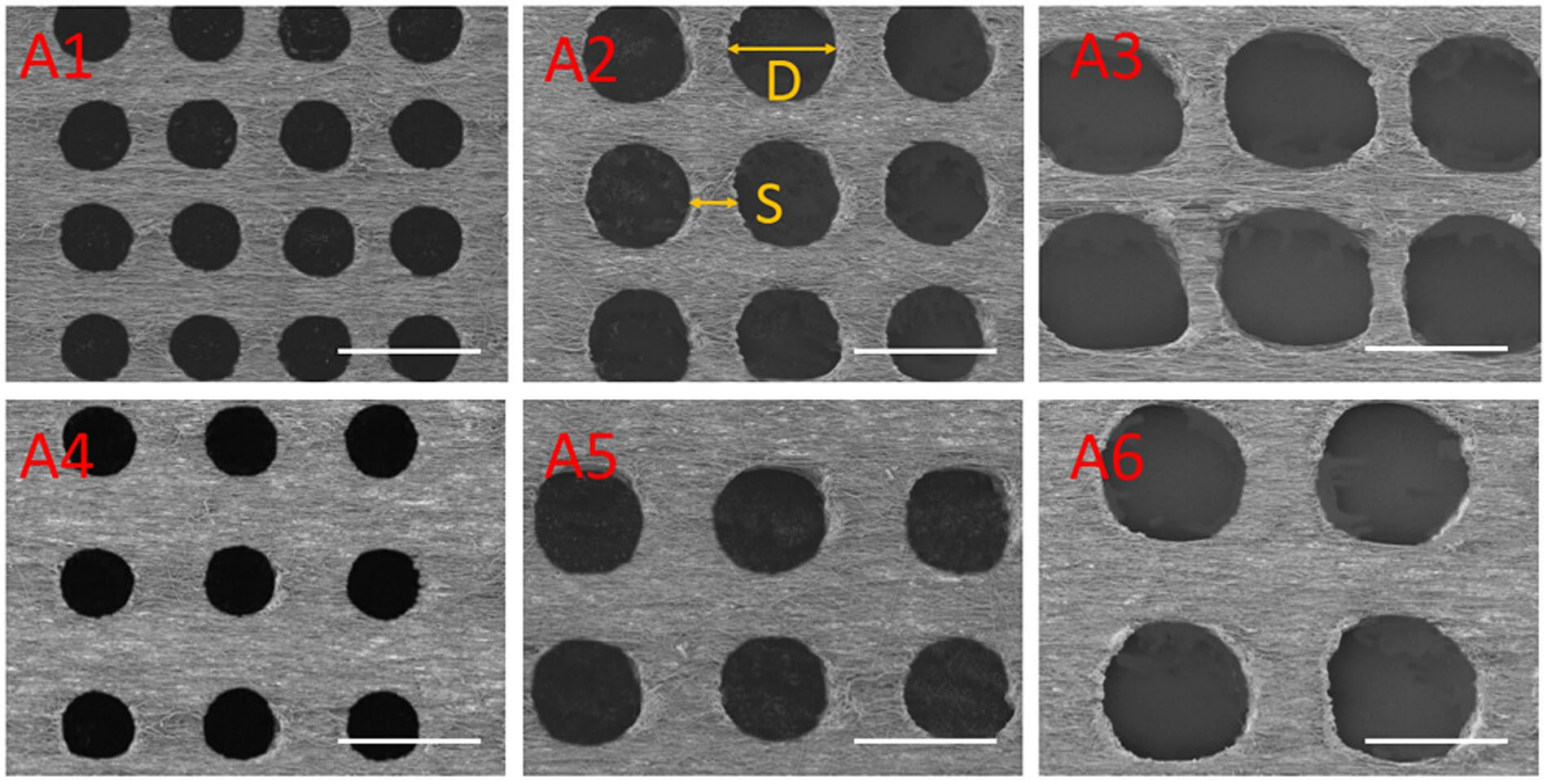
SEM images of laser-perforated, aligned PLGA scaffolds with different hole sizes (D) and spacings (S); A1-A6 are the samples shown in Table 1. The scale bar is 200 μm.

After the formation of the aligned fibrous scaffolds, a picosecond laser machine was used to ablate macro-scale holes in the constructs. By controlling the laser parameters, the holes in six samples shown in Table 1 were made with minimal thermal stress and collateral damage, as shown in Fig. 3. “D” represents the diameter of the perforated holes, and “S” represents the spacing, i.e., the distance between holes.

**Table 1:** Six samples with different hole diameters and spacings.

Samples	A1	A2	A3	A4	A5	A6
D-S (μm)	100–50	150–50	200–50	100–100	150–100	200–100

**Figure 4:**
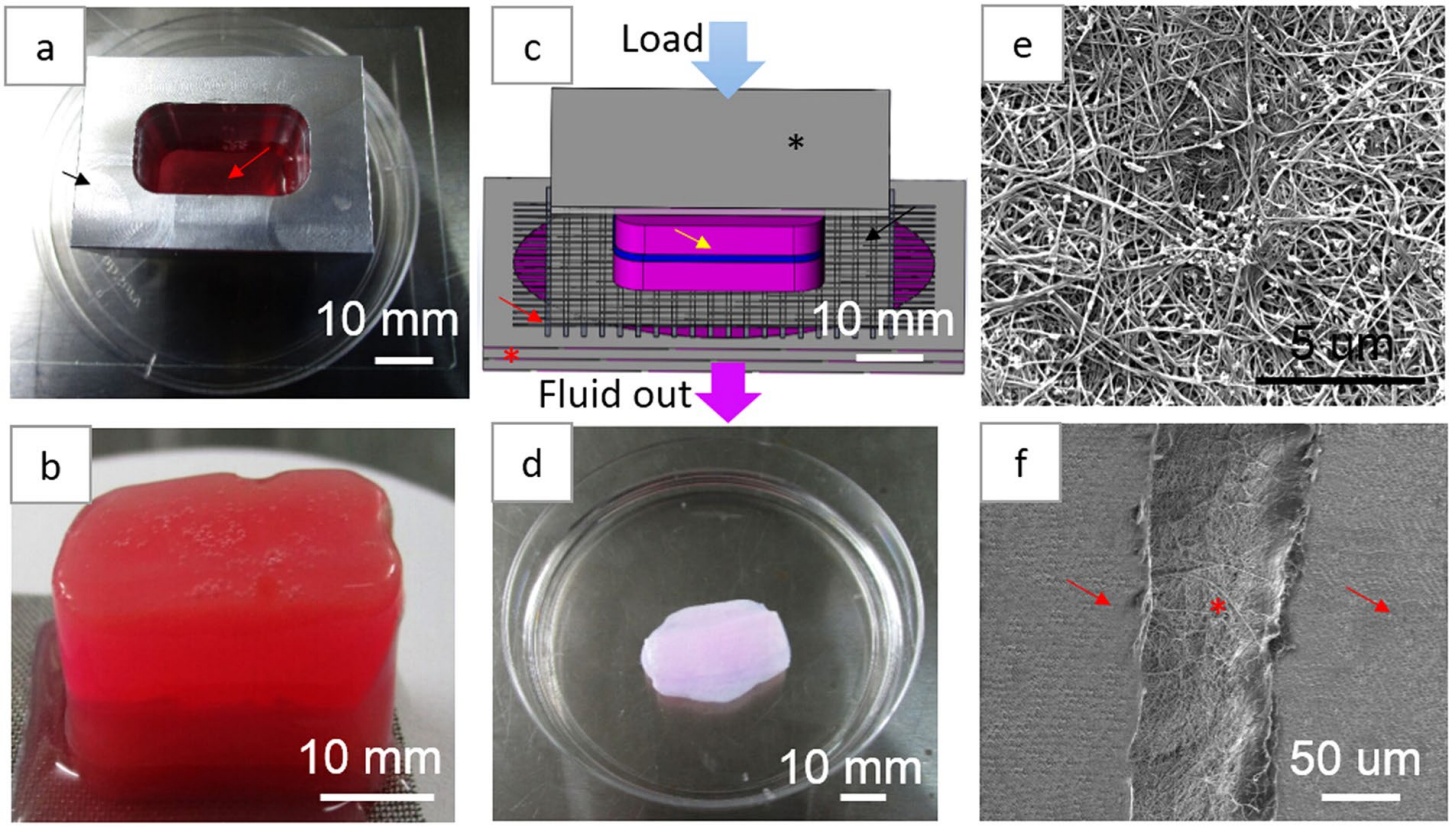
The hybrid scaffold production process: (**a**) PC collagen mold (black arrow) and collagen hybrid solution (red arrow); (**b**) hybrid scaffold construct gel; (**c**) schematic of the compression process of hybrid gel (yellow arrow) with glass plate (black asterisk), nylon mesh (black arrow), stainless steel mesh (red arrow) and filter papers (red asterisk); (**d**) hybrid scaffold; (**e**) SEM image of the hybrid scaffold surface; and (**f**) the oblique section of the hybrid scaffold, showing the three-layered structure with electrospun mat (red asterisk) and collagen layers (red arrow).

Figure 4a–d shows the main process used to produce the hybrid constructs consisting of perforated electrospun membranes combined with PC collagen. Figure 4a shows the mold (black arrow). After the aligned, electrospun PLGA mat was placed on the first collagen layer (Fig. 4a, red arrow), another 5 mL of collagen mixture solution was poured into the mold to form a second layer of collagen. Figure 4b shows the hybrid construct gel that formed after incubating the material shown in Fig. 3a at 37 °C for 30 min. Figure 4c is a schematic of the compression process. The construct was subjected to unconfined compression by a 120 g weight for 5 min, yielding the hybrid construct (Fig. 4d). Figure 4e is an SEM image of the hybrid construct surface; only compressed collagen can be observed because the PLGA mat was in the middle of the construct. By imaging the oblique section of the construct, the three layers could easily be observed; the electrospun mat (Fig. 4f, red asterisk) in the middle was tightly adhered to the collagen layers (red arrow, Fig. 4f).

**Figure 5:**
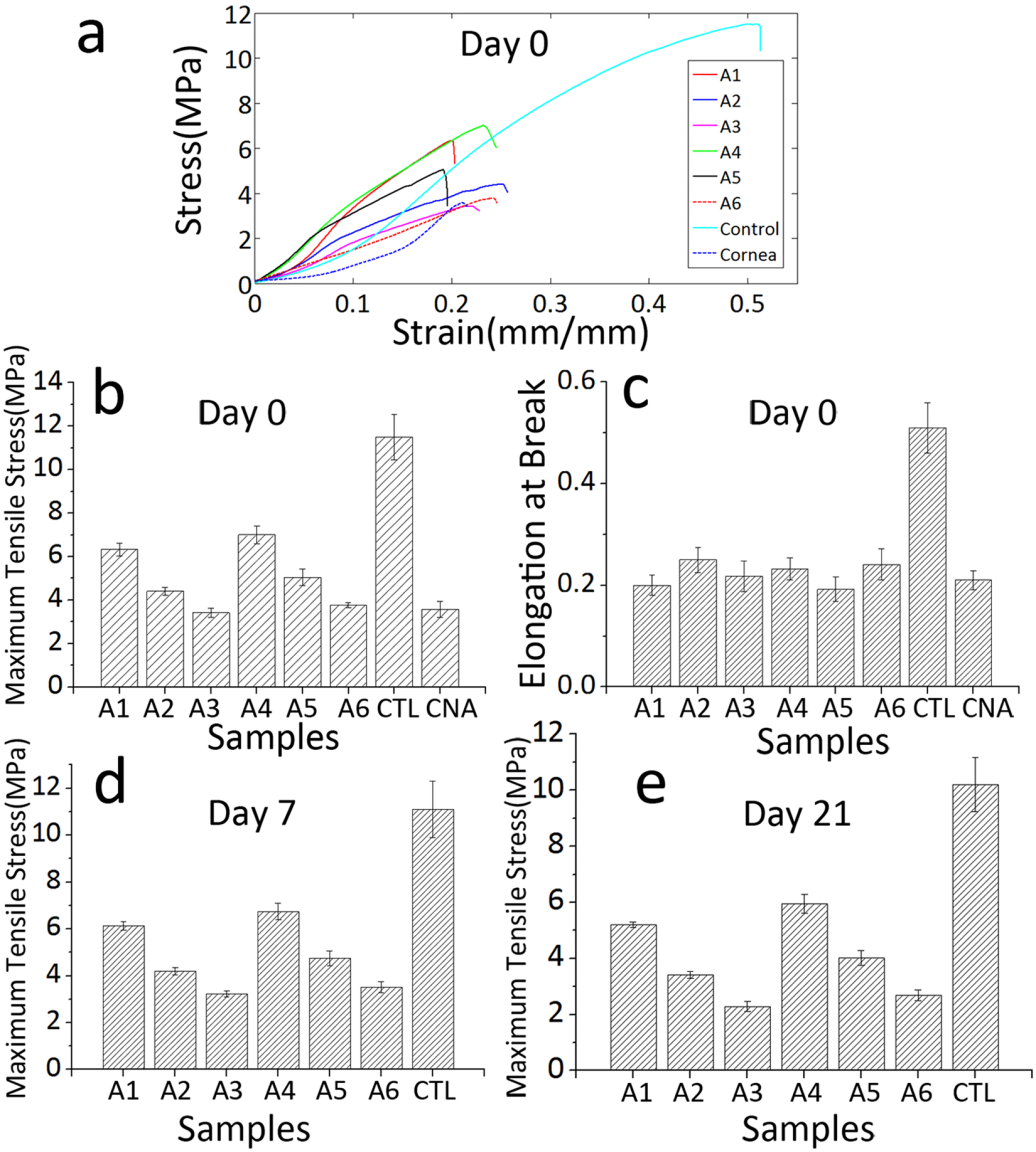
Mechanical properties: (**a**) Average strain-stress curves of hybrid constructs A1-A6, the control group and the acellular pig cornea after incubating in PBS for 1 h; (**b**) maximum tensile stress histogram of the samples in (**a**), CTL is the control group and CAN is the acellular pig cornea; (**c**) histogram of strain at break of the samples in (**a**); histogram of maximum tensile stress of hybrid constructs A1-A6 and the control group after incubating in PBS for 7 days (**d**) and 21 days (**e**).

The mechanical properties and light transmittance of the six hybrid samples (in the following content, the six hybrid samples denoted as A1-A6 were PC collagen-PLGA hybrid constructs with perforated electrospun mats A1-A6, respectively) were measured to identify the optimal hole size and spacing, with which the hybrid constructs would exhibit properties similar to those of native corneal tissue. Figure 5a shows the strain-stress curves obtained from the INSTRON testing machine after incubating the samples in PBS for 1 h, the maximum tensile stress of these hybrid constructs is shown in Fig. 5b and c shows a histogram of elongation at break data. Figure 5d and e are the maximum tensile stress of these hybrid constructs after incubating in PBS for 7, 21 days, respectively. The control was the hybrid constructs that were not perforated, which exhibited a maximal tensile strength of 11.5 ± 1.05 MPa. The maximum tensile strength of acellular cornea was 3.57 ± 0.36 MPa and the strain at break was 0.21 ± 0.02, which were in accordance with the results reported before that the tensile strength of natural corneal tissue was approximately 3–5 MPa and strain at break was approximately 0.192^52^. We found that the laser perforation could be used to reduce the tensile strength to make the hybrid construct with tensile strength more similar to natural cornea. When hole diameter was kept constant, tensile strength decreased with decreasing hole spacing, and when hole spacing was kept constant, tensile strength also decreased with increasing hole diameter. Thus laser perforation could improve the mechanical properties of the hybrid constructs for corneal tissue engineering applications. The elongation at break was linear, independent of hole size and spacing, as shown in Fig. 5c. The maximum elongation of the constructs was reduced through laser perforation, rendering it more similar to that of the natural human cornea. Through the contrast of Fig. 5b,d,e, we could find out with the increase of incubating time, the maximum tensile stress of all constructs decreased gradually.

A microplate reader was utilized to determine the light transmittance of the six hybrid construct samples, the control and acellular cornea under wavelengths ranging from 400 to 750 nm. We tested the light transmittance of the samples after 0, 1, 3, and 7 days of immersion in PBS. The absorbance (A) data were obtained directly from the microplate reader, and the light transmittance (T) data were calculated from the formula: %T = 10^2−A^.

**Figure 6:**
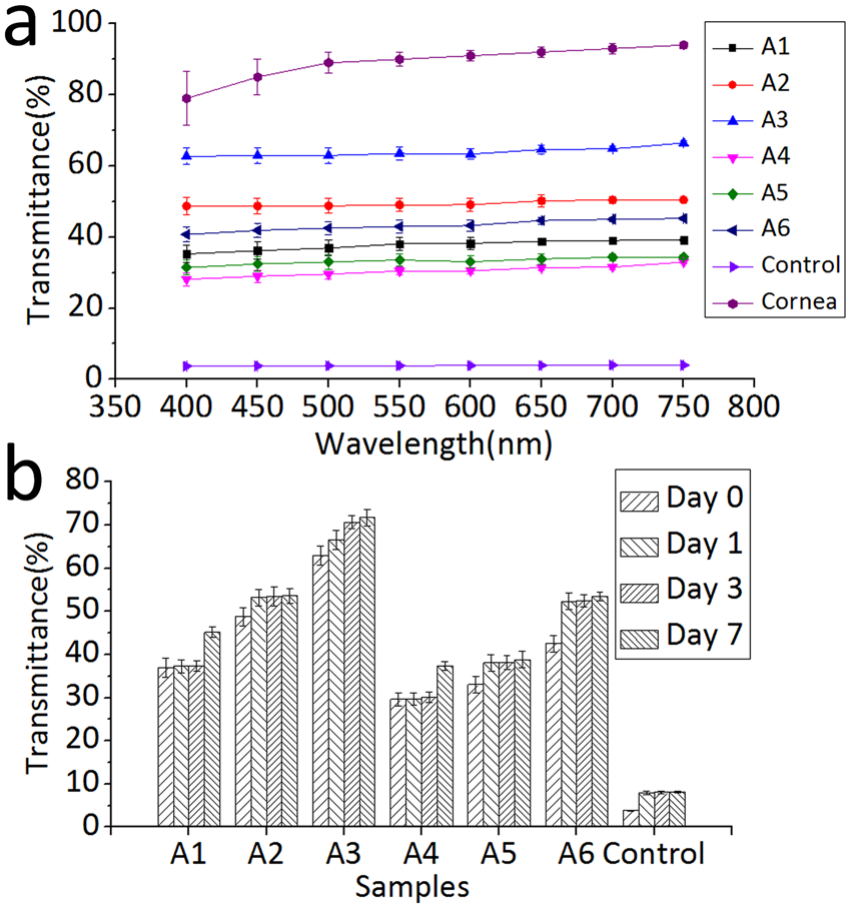
The transparency test results; (**a**) The average transmittance spectra of hybrid constructs A1-A6, the control group and the acellular pig cornea after incubating in PBS for 1 h (day 0); (**b**) Transmittance histogram of six hybrid constructs A1-A6 and the control after 0, 1, 3, and 7 days of immersion in PBS under the 500 nm wavelengths.

The average light transmittance data of samples after immerged into PBS for 1 h (day 0) are shown in Fig. 6a. Transmittance of six hybrid constructs A1-A6 and the control after 0, 1, 3, and 7 days of immersion in PBS under the 500 nm wavelengths are shown in Fig. 6b. For natural cornea, the transmittance under 500 nm wavelength is up to 90%. For constructs A1-A6 and the control, the resulting trend observed between different samples indicated that when the hole diameter was the same, light transmittance decreased with increasing hole spacing; when the hole spacing was the same, light transmittance increased with increasing hole diameter. The resulting trend observed in one sample over time indicated that the hybrid construct transparency increased with increasing time.

Taken together, the tensile strength and light transmittance results of the six hybrid samples with different hole sizes and spacings indicated that the properties of the hybrid sample A3 (200–50 μm) (maximum tensile stress 3.42 ± 0.22 MPa, transmittance at 500 nm wavelength 63 ± 2.17%) were most suitable to apply in corneal tissue engineering. In the following content, the term “laser-perforated hybrid construct” refers to that perforated with 200–50 um holes by the picosecond laser, and the control was not perforated.

### Live/dead staining

**Figure 7:**
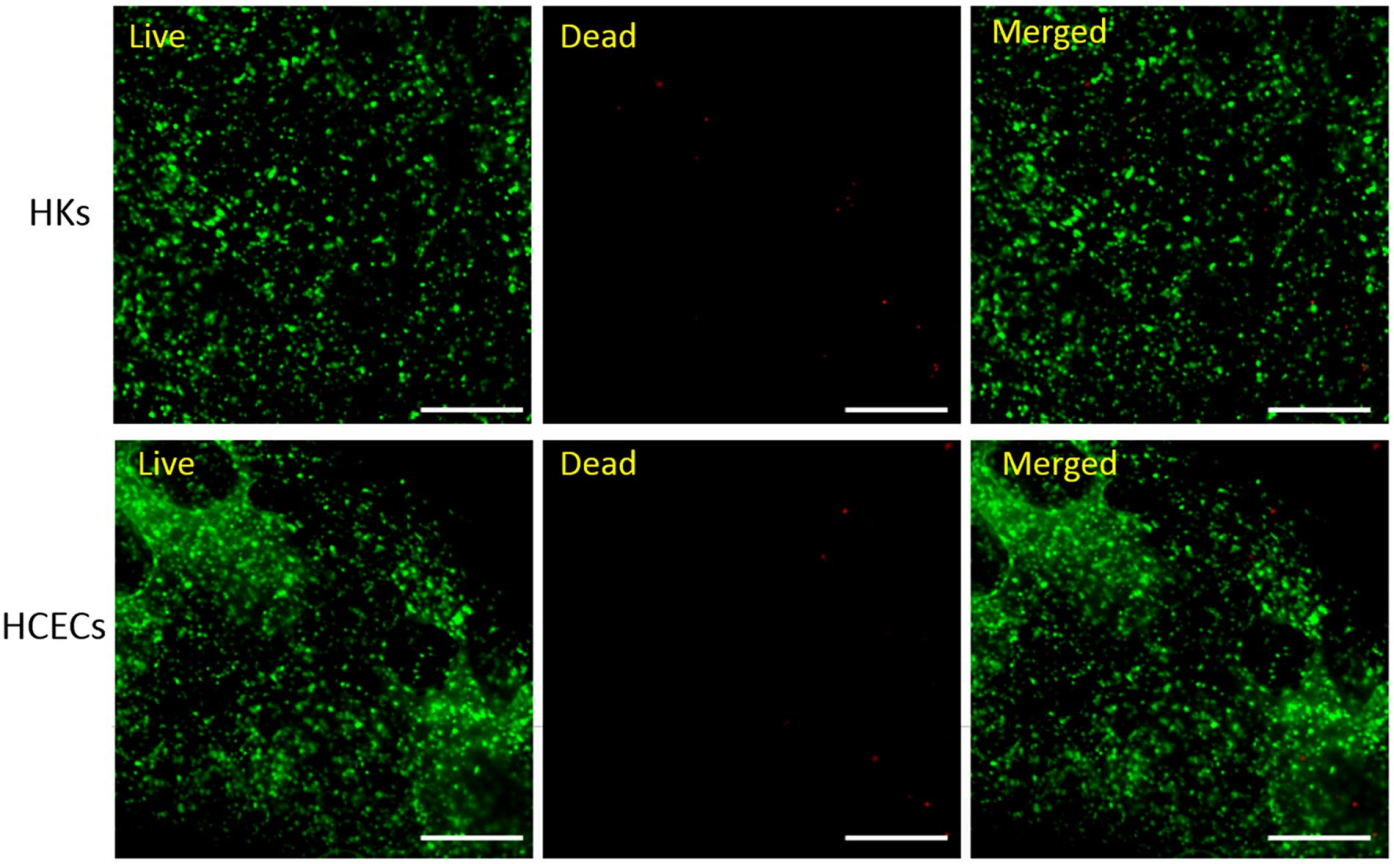
HKs and HCECs viability on the hybrid constructs. The scale bar is 500 μm.

As shown in Fig. 7, there was minimal HKs death caused by the compressing process, and we could also discover that the HCECs cells adhered on the construct well and kept high viability. Thus, the hybrid materials and fabrication method used in this experiment did not have any harmful effect on the encapsulated cells and could support cells viability without disruption of constructs.

### Cell proliferation assay

**Figure 8:**
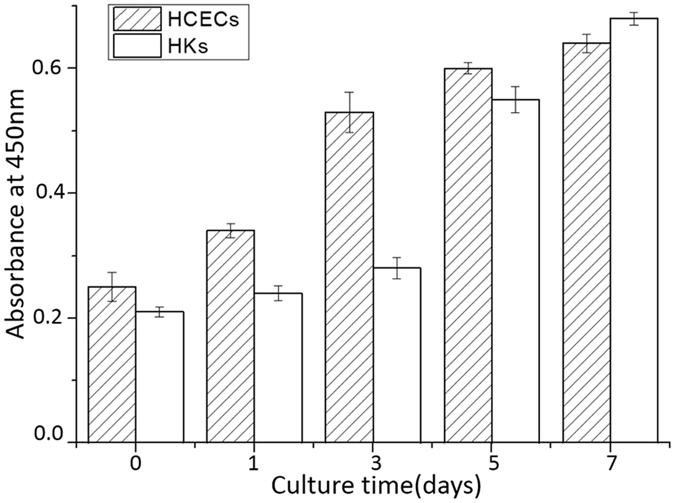
Proliferation of HKs and HCECs on the hybrid constructs within 7 days.

Moreover, the proliferation of HCECs and HKs were qualitative determined by OD value at 450 nm (Fig. 8). From the histogram image, it could be find out that the number of HCECs had a fast increase before culturing for 3 days and from day 3 to day 7, the increase rate reduced. For HKs, the number of cells increased slowly before 3 days culture, from day 3 to day 5 it had a large increase and after day 5, the increase rate also reduced. Although having different growing mode, both cells proliferated well, whether encapsulating within the hybrid construct or inoculating on the surface of the construct.

### Immunohistochemistry of HKs and HCECs on the construct

**Figure 9:**
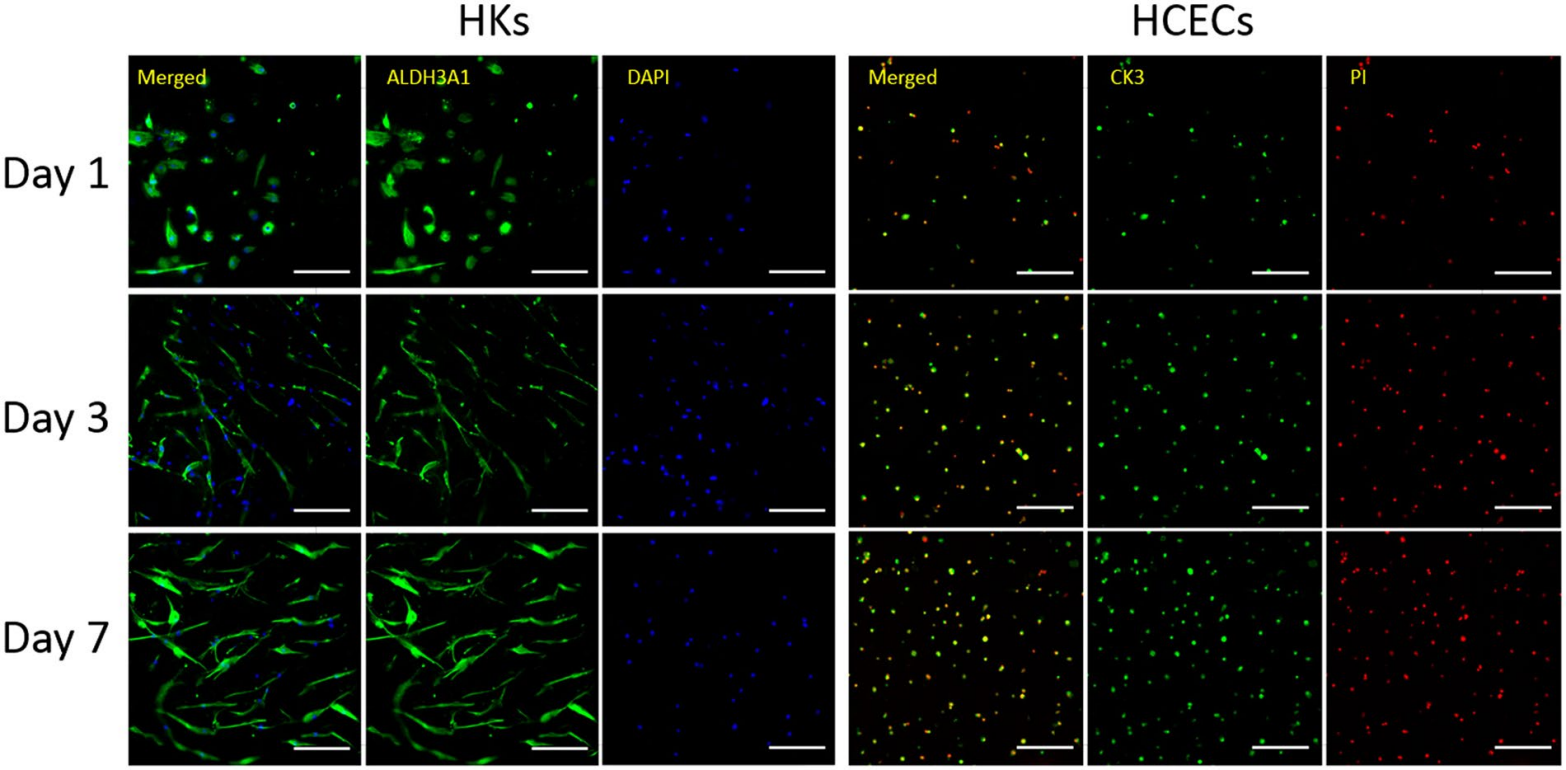
Protein expression of specific markers in HKs within hybrid scaffolds. Images (left) show the fluorescent staining of ALDH3A1 (green) and nuclei (blue) in HKs after being cultured for 1, 3, and 7 days. Protein expression of specific markers in HCECs seeded on the surface of hybrid scaffolds. Images (right) show fluorescent staining of CK3 (green) and nuclei (red) in HCECs after being cultured for 1, 3, and 7 days. (scale bar, 200 μm).

**Figure 10:**
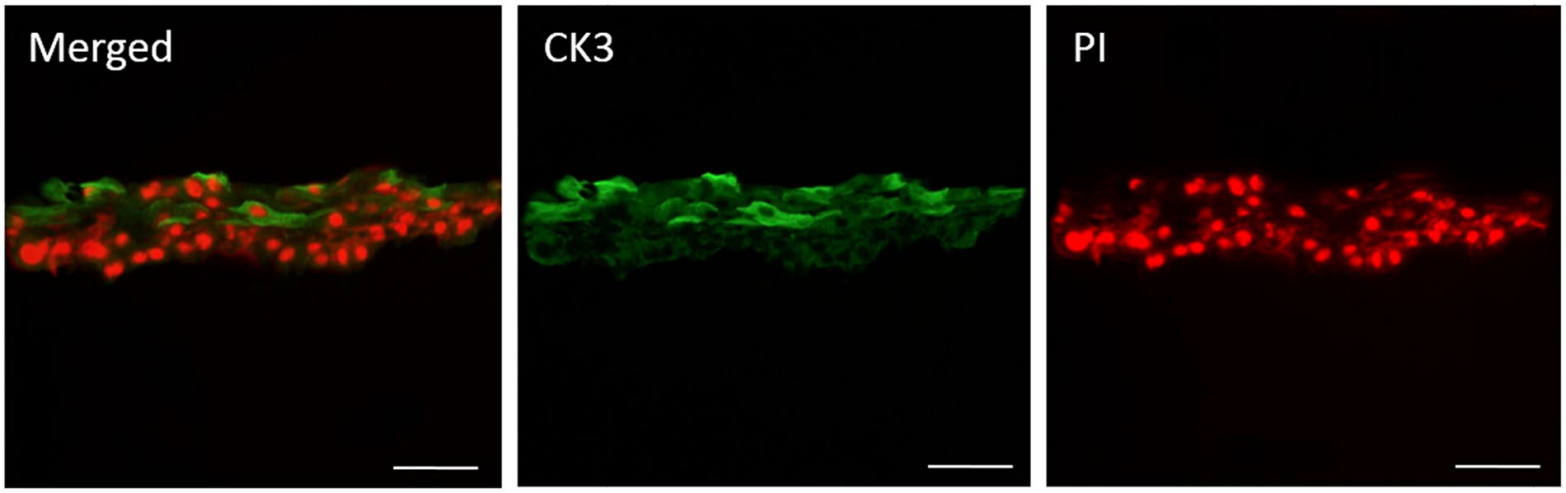
Fluorescent staining of histological cross sections of HCECs on the surface of hybrid constructs. Images show CK3 (green) and nuclei (red) in HCECs after culturing for 2 weeks. (scale bar, 100 μm).

After 1, 3, and 7 days of culture, the localization and expression of ALDH3A1 in HKs and CK3 in HCECs were investigated by confocal microscopy. As Fig. 9 shows, the HKs and HCECs proliferated during the culture periods. Compared with day 1, the numbers of both cells were higher on days 3 and 7, indicating that the laser-perforated hybrid construct were biocompatible and that the cells within or on the surface of the constructs were proliferating well. Additionally, the shape of the HKs started to change from round to dendritic morphology after day 3. After another 7 days air-lifting culture, the HCECs could form multi layers on the surface of construct by fluorescence staining of frozen section, as shown in Fig. 10, and CK3 was strongly expressed in superficial cell layers.

## Discussion

As the native cornea mainly consists of collagen fibers, electrospinning can easily be used to obtain aligned collagen fibrous constructs that mimic the corneal stroma; similarly, disorganized constructs can be used to mimic epithelium^1, 53^. However, the highly hydrophilic nature of collagen makes it structurally weak such that it needs to be cross-linked by chemical agents, which may influence cells seeded on the material^54, 55^. The mechanical properties of the scaffold can be improved by mixing collagen with synthetic polymers prior to being electrospun; however, crosslinking would still be needed. Furthermore, the same solvent must be used for the polymers and the collagen to avoid the split-phase; thus, the application of this method is limited^55^. Another technique is the plastic compression of the collagen solution. While the constructs obtained by this method do not require crosslinking, the resulting mechanical properties are also weak with a tensile strength of approximately 0.6 MPa^34^. However, by combining the two techniques, their disadvantages can be overcome. First, a wide variety of materials can be used for electrospinning. Second, the combination allows the mechanical properties to be greatly improved without crosslinking. Meanwhile, as collagen forms both the top and bottom layers, the hybrid constructs also exhibit excellent biocompatibility.

To make a construct with mechanical properties similar to natural cornea, we put forward to use laser perforation to create macro-sized holes for the regulation of tensile strength. The reason for choosing holes and rectangular array pattern is that this is easy to be realized and can be well controlled to obtain the optimal result when comparing with other more complicated shapes and patterns. We have roughly pre-tested the effect of hole sizes and spacings on mechanical properties and transparency, and then choose a rational size range, shown in Table 1, to find out the optimal sizes and spcings. Rather than laser perforating the whole hybrid construct, electrospun PLGA membrane was first perforated by a picosecond laser and then combined with compressed collagen to form a hybrid construct. On the one hand, this sequence avoids damaging the HKs in the compressed collagen with the laser. On the other hand, the three membranes adhere more tightly by the permeation of collagen gel into the holes, yielding a compressed hybrid construct with a higher structural integrity, demonstrated by Supplementary Figs 1 and 2. The influence of hole size and spacing on tensile strength can be easily observed from the data shown in Fig. 5a,b. And these results are in accordance with those reported by Benjamin Li-Ping Lee *et al*.^38^. The possible reason would be that with either increasing hole size or decreasing hole spacing, the area of the holes will increase; in essence, this means that the connection area that supports the whole scaffold will decrease, resulting in decreased tensile strength. The elastic modulus of the hybrid constructs was also calculated, and the elastic modulus was found to be independent of hole sizes and spacings because the elongation at break only changed with the process of laser perforation rather than the perforation sizes and patterns. Perhaps the elastic modulus is relative to the alignment degree of the electrospun mat within the hybrid construct, the mechanism of which was studied by Akhilesh K Gaharwar *et al*.^56^. Although the mechanical properties of all constructs are decreasing gradually over time, the lowest tensile stress among all these samples is 2.1 MPa after incubating in PBS for 21 days, which means our construct has a long-term stable mechanical property. The reason for choosing aligned electrospun rather than non-aligned one is that we have already determined the mechanical properties of aligned and non-aligned electrospun mat under the same conditions and found that the mechanical properties of non-aligned mat (9.36 ± 0.88 MPa) were lower than that of the aligned mat (11.5 ± 1.05 MPa). Thus on the basis of final mechanical properties similar to that of natural corneal tissue, the aligned electrospun mat can give a less compromise to the light transmittance than non-aligned one. That means with aligned electrospun mat, the hybrid construct can have a better light transmittance (optimal 63 ± 2.17% under 500 nm wavelength), than that with non-aligned mat (optimal 40 ± 1.8% under 500 nm wavelength), when the mechanical properties of both mats are similar to the natural corneal tissue. In addition, our hybrid construct has a wide applications in tissue engineering when the biocompatibility and mechanical properties need to be considered together.

Light transmittance is an important parameter for corneal tissue, and it is essential to consider this issue when constructing a tissue-engineered corneal scaffold. Conventionally, the light transmittance of electrospun constructs has mainly been improved by varying the choice of materials in favor of natural materials, such as collagen, gelation, chitosan and silk fibroin^57–60^. However, these natural materials either are difficult to electrospin into fibers with desirable morphology or the electrospun fibrous constructs exhibit poor mechanical properties. While these limitations necessitate the incorporation of other synthetic polymers, it is a complex challenge to identify suitable materials and optimal blend ratios^57, 58^. In this study, we put forward a novel method to improve the light transmittance of electrospun constructs, which utilizes a picosecond laser to ablate macro-scale holes. In this way, the light transmittance is mainly determined by the size of and the space between the perforated holes instead of electrospinning material, which makes it easier to improve the light transmittance. The result indicated that the larger the hole size, the smaller the spacing between holes, the better the light transmittance. The reason for this phenomena would be that with larger holes and smaller spacings, the areas that the light can directly get through are larger, which results in higher transmittance. And the light transmittance increases with increasing time of incubating in PBS. This may because over time, the electrospun PLGA mat begins to slowly degrade, and degradation occurs to a greater extent around holes where the fiber connections are not tight and firm, demonstrating by the gradual reduction of mechanical property of the hybrid construct. With the degradation, hole size is gradually increasing and fibers are gradually loosening, both of which can result in increased light transmittance. This can also explain the result that the mechanical properties of constructs decreasing gradually over time. These results indicate that the constructs will degrade over time, allowing new tissues to grow gradually; throughout this process, the light transmittance will drastically increase until finally reaching the required level. In combination with the observed mechanical properties, these results indicated that the optimal hole size and spacing when applied in corneal tissue engineering was 200–50 μm.

To assess the biocompatibility of the optimal construct, the adhesion and proliferation of cells on the construct were studied. HKs were encapsulated within the PC collagen, and HCECs were seeded on the surface of the hybrid construct, simulating the structure of the natural cornea (i.e., the relative location of the epithelial and stromal layers). Viability staining and CCK-8 results of the two cells demonstrated that both cells displayed a high survival rate *in vitro* culture conditions. This supports the idea that the hybrid construct is a non-toxic and biocompatible materials. CCK-8 assay showed that both cells were metabolically active and proliferated well.

Cell-specific protein expression was used for cell definition and cell activity analyses. CK3 is specifically expressed in HCECs and is often recognized as a marker of HCECs^61^. ALDH3A1 is specifically expressed in HKs and is often recognized as a marker of HKs^62, 63^. The number of ALDH3A1-stained and CK3-stained cells increased from day 1 to day 7 of culture, indicating that the HKs and HCECs could normally proliferate, synthesize and strongly express proteins within and on the surface of the hybrid constructs, respectively. Additionally, the shape of HKs changed from round to dendritic morphology during the culture within the hybrid construct, which is a typical shape for HKs *in vivo* ^64–66^. The change of shape mainly because the cells keep the round shape which is a typical shape after digestion, after culturing for several days, the cells begin to recover their typical spindly shape and proliferate normally. Frozen section staining indicates that 7 days of airlifting is sufficient to allow HCECs stratification to a multilayered epithelium.

## Conclusions

In this study, electrospinning and PC collagen were combined to fabricate sandwich-like hybrid constructs with good biocompatibility and mechanical properties. Macro-scale holes with different sizes and spacings were laser-perforated in the electrospun PLGA mats, and through the determination of mechanical properties and light transmittance of the hybrid constructs, the optimal hole size and spacing of 200–50 μm was identified. These parameters yielded a perforated hybrid construct with properties that were similar to those of the native cornea. HKs and HCECs were cultured within and on the surface of the optimal hybrid construct for 2 weeks, both cell types exhibited good adhesion and proliferation and maintained their respective phenotypes well, and HCECs could form multi layers. These findings demonstrate that our hybrid construct is suitable for constructing engineered corneal tissue.

## Electronic supplementary material

Supplementary
