# Supplementary material for: Tissue-engineered cornea constructed with compressed collagen and laser-perforated electrospun mat
Source: Sci Rep. 2017 Apr 20;7:970. doi: 10.1038/s41598-017-01072-0 (PMC5430529; doi:10.1038/s41598-017-01072-0)
Supplement: Supplementary file 1 — Supplementary [file 41598_2017_1072_MOESM1_ESM.pdf]

Supplementary Materials for:

Tissue-engineered cornea constructed with compressed collagen and laser- perforated electrospun mat

Bin Kong, Wei Sun, Guoshi Chen, Song Tang, Ming Li, Zengwu Shao, Shengli Mi*

*Corresponding author: E-mail address: mi.shengli@sz.tsinghua.edu.cn

We firstly perforated the aligned, electrospun PLGA membrane by a picosecond laser and then combined it with compressed collagen to form a hybrid construct. Thus the three membranes adhered more tightly by the permeation of collagen gel into the holes. This result was demonstrated by shear strength test. We established one model shown in Fig. 1, a) is the control group, the hybrid construct is consist of two PC collagen mats (red arrow) and one PLGA electrospun membrane (yellow star) without holes; b) is the experimental group, the PLGA membrane was firstly perforated by picosecond laser and then combined by two PC collagen gels and the compression process will allow the collagen gels to permeate into holes (green arrow); c) is the perspective image of b), we can see the through-holes on the electrospun membrane and d) shows the test method, we use the INSTRON tensile machine to put force on both side of the PC collagen and when the construct is broken, the force F will be the maximum tensile force or shear strength the construct can bear. Fig. 2 is the histogram image of maximum shear strength of the two construct a) and b). We can find out that the shear strength which the construct with perforated holes can bear is much larger than that without holes. Thus we can conclude that the three membranes will adhere more tightly by the permeation of collagen gel into the holes.

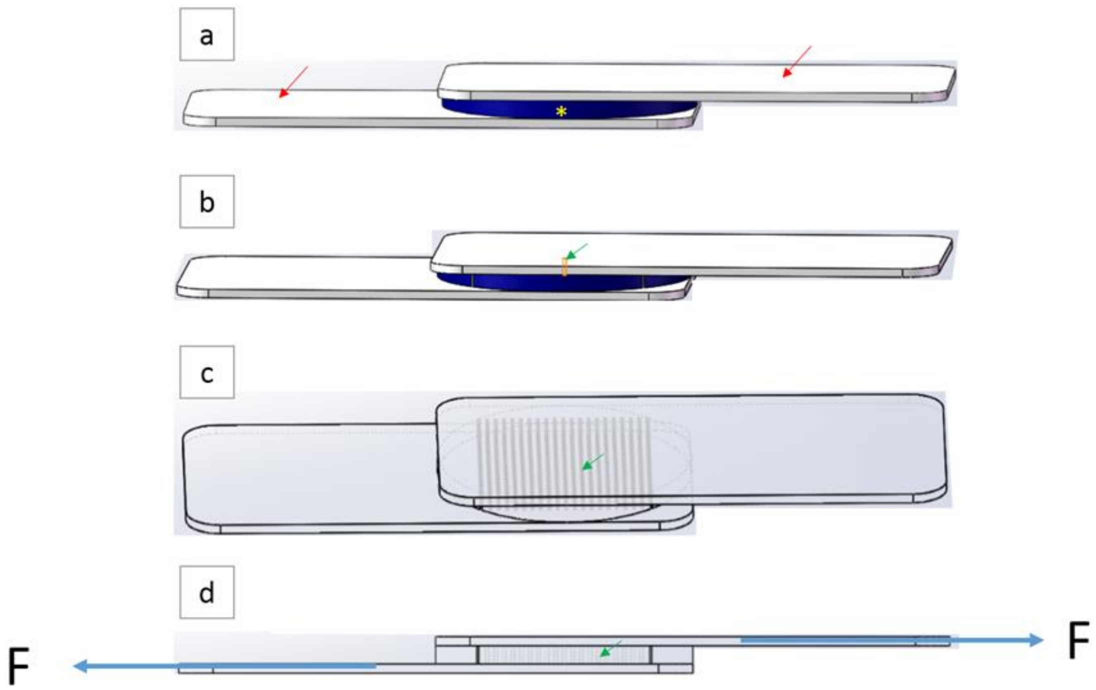

Fig. 1. The test model, a) is the control group, the hybrid construct is consist of two PC collagen mats (red arrow) and one PLGA electrospun membrane (yellow star) without holes; b) is the experimental group, the PLGA membrane was firstly perforated by picosecond laser and then combined by two PC collagen gels and the compression process will allow the collagen gels to permeate into holes (green arrow); c) is the perspective image of b) and d) is the test method, with force F exerting on both side of the PC collagen.

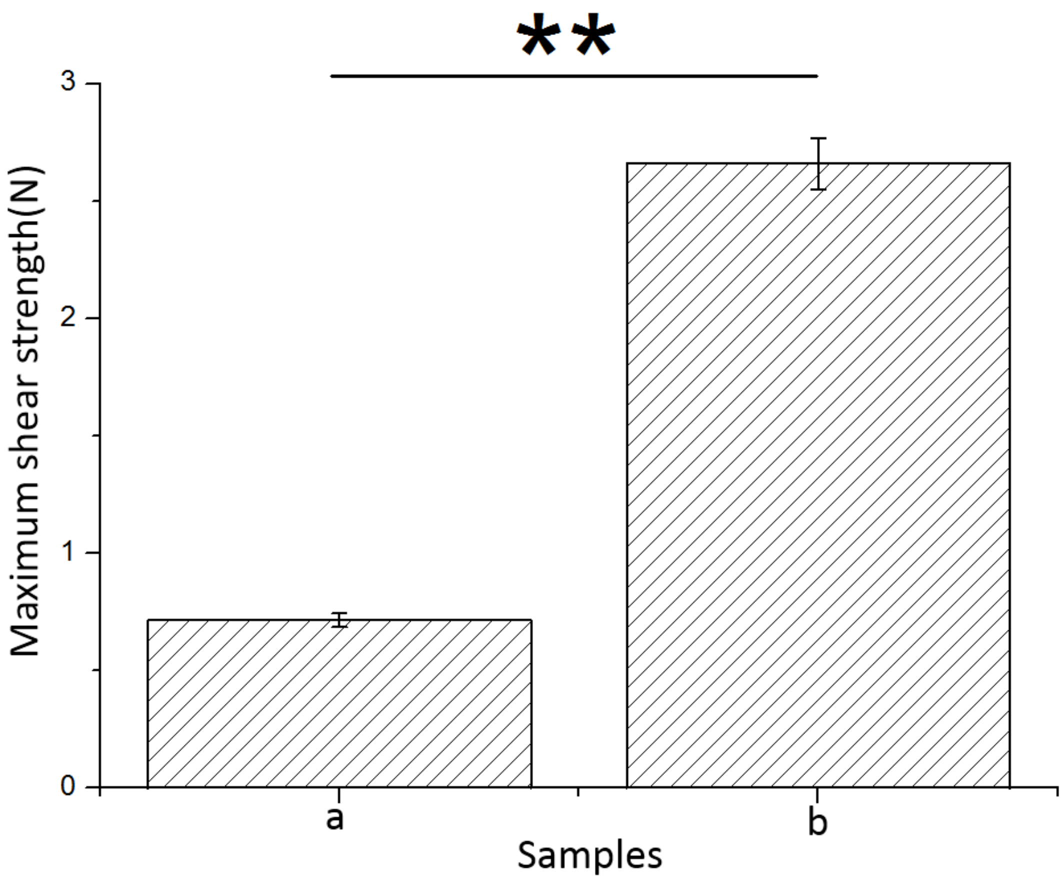

Fig. 2. The histogram image of maximum shear strength of the two construct a) and b) in Fig. 1. Data represent means ±SD (**p < 0.01)
